# Disentangling the intertwined roles of mutation, selection and drift in the mitochondrial genome

**DOI:** 10.1098/rstb.2019.0173

**Published:** 2019-12-02

**Authors:** Sarah Schaack, Eddie K. H. Ho, Fenner Macrae

**Affiliations:** Department of Biology, Reed College, Portland, OR 97202, USA

**Keywords:** mutation rate, heteroplasmy, effective population size, genetic bottleneck, mutation accumulation, mtDNA

## Abstract

Understanding and quantifying the rates of change in the mitochondrial genome is a major component of many areas of biological inquiry, from phylogenetics to human health. A critical parameter in understanding rates of change is estimating the mitochondrial mutation rate (mtDNA MR). Although the first direct estimates of mtDNA MRs were reported almost 20 years ago, the number of estimates has not grown markedly since that time. This is largely owing to the challenges associated with time- and labour-intensive mutation accumulation (MA) experiments. But even MA experiments do not solve a major problem with estimating mtDNA MRs—the challenge of disentangling the role of mutation from other evolutionary forces acting within the cell. Now that it is widely understood that any newly generated mutant allele in the mitochondria will initially be at very low frequency (1/*N*, where *N* is the number of mtDNA molecules in the cell), the importance of understanding the effective population size (*N*_e_) of the mtDNA and the size of genetic bottlenecks during gametogenesis and development has come into the spotlight. In addition to these factors regulating the role of genetic drift, advances in our understanding of mitochondrial replication and turnover allow us to more easily envision how natural selection within the cell might favour or purge mutations in multi-copy organellar genomes. Here, we review the unique features of the mitochondrial genome that pose a challenge for accurate MR estimation and discuss ways to overcome those challenges. Estimates of mtDNA MRs remain one of the most widely used parameters in biology, thus accurate quantification and a deeper understanding of how and why they may vary within and between individuals, populations and species is an important goal.

This article is part of the theme issue ‘Linking the mitochondrial genotype to phenotype: a complex endeavour’.

## 1. The importance of estimating mtDNA mutation rates and our current state of knowledge

Knowing the rate of mutation in the mitochondrial genome (mtDNA) of eukaryotes is a key parameter in biological research, for a range of basic and applied questions. On one end of the spectrum, mtDNA mutation rates (MRs) are, for example, essential components of efforts to investigate patterns of molecular evolution (e.g. calibrating molecular clocks; [1]). On the other end of the spectrum, mtDNA MRs are important for estimating disease risk, rates of ageing and the likelihood of rapid adaptation to a changing climate [2–4]. Regardless of the question to which rates are applied, the general idea is that knowing the rate of mutation can provide some insight into the amount of genetic variation being introduced into a population, upon which evolutionary forces might then act. Without an estimate of the MR, larger questions about rates of diversification, the significance of highly conserved regions of the genome and the evolution of populations are difficult to pursue.

Most estimates of mtDNA MRs are based on the so-called indirect or phylogenetic methods, where the number of nucleotide differences in (putatively) neutral regions of the genome is counted in two lineages with a known divergence time. The number of changes observed is then divided by the number of possible loci and time (either absolute time or number of generations) in order to calculate a rate. This is not, in fact, an estimate of the MR, but is instead an estimate of the *substitution rate*—but the distinction is rarely made in the general scientific literature. While the substitution rate can be a good approximation of the MR if substitutions are neutral, perhaps unsurprisingly, the substitution rate can be much different than the MR depending on the impact selection on the mutations generated by faulty polymerase function or unrepaired DNA damage. In cases where substitution rates and MRs have both been estimated for the same species, substitution rates tend to be far lower than MRs, presumably because the number of mutations lost to natural selection is not accounted for by indirect methods [5].

In contrast to indirect methods, direct methods of MR estimation depend on either mutation accumulation (MA) studies or trio sequencing [6,7]. The difference between estimates from indirect and direct methods is the emphasis on eliminating selection. Both methods assume selection has been minimized somehow—in MA studies, for example, this is achieved by propagating lineages from a known ancestor via single-progeny descent in non-stressful laboratory conditions. Minimizing selection with a genetic bottleneck of one individual every generation means that, even if mutations that occur are deleterious, they can be passed on to the next generation and can be part of a tally used to estimate the MR. In taxa where MA studies and indirect methods have both been used, for example, *Caenorhabditis elegans*, the differences in the rate estimates are at least an order of magnitude higher when directly observed [8].

**Figure 1.**
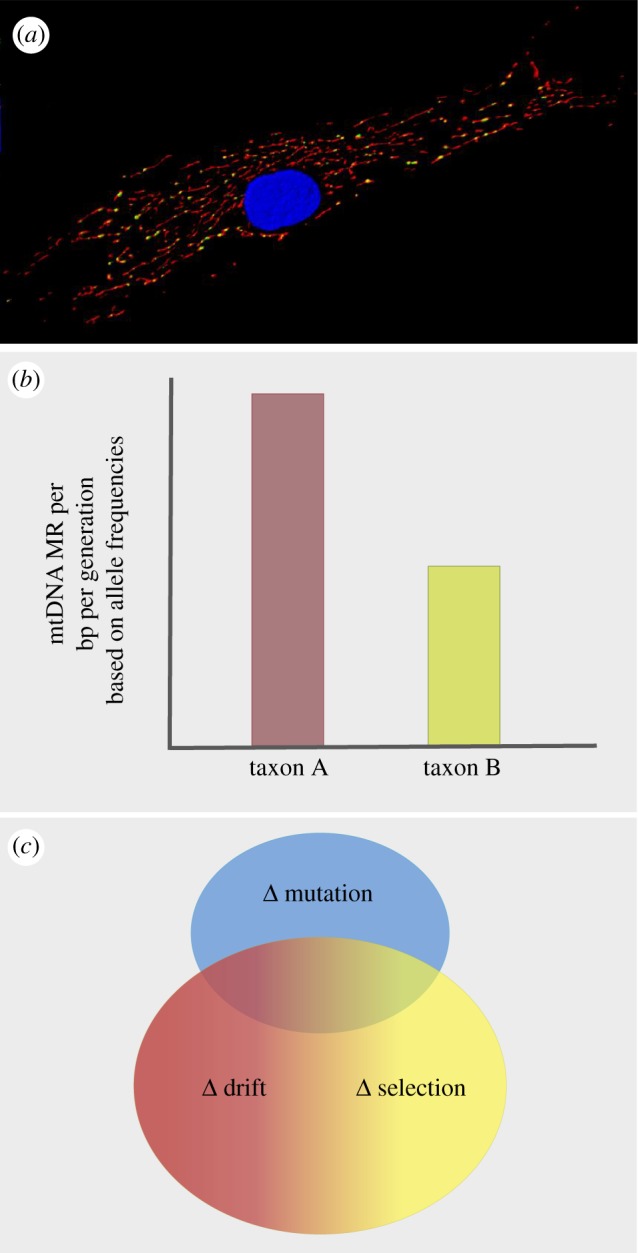
(*a*) Photo of a human fibroblast. The nucleus (blue) is surrounded by the mitochondrial matrix (red) which contains the numerous mtDNA nucleoids (stained green, but appear yellow because of the overlay). Heteroplasmy (more than one version of the mtDNA in a particular cell) can be the result of mutation (faulty DNA replication or unrepaired DNA damage) or biparental inheritance of mtDNA. Photo credit: Amanda Bess/Joel Meyer. (*b*) Mutation rate estimates, which can differ among genotypes, populations and species, are based on sequencing the genome and counting the frequency of new, mutant mtDNA alleles in either mutation accumulation lines or parent-offspring trios. (*c*) While apparent differences in mtDNA MRs illustrated in (*b*) could be explained by differences in MRs (top circle; blue), changes in mtDNA allele frequencies over time can also be the result of genetic drift and intracellular selection—two inversely correlated evolutionary forces (bottom circle; red and green)—which also can shape allele frequencies in heteroplasmic lineages and obscure the estimation of the MR.

Since the first direct estimates of mitochondrial MRs were reported, only a few other species have been studied (see [9] for a recent review). This paucity of mtDNA MR estimates is owing, in large part, to the practical and ethical constraints on which taxa can be subject to MA and whole-genome sequencing, but there will probably be a surge in the data available as the price of sequencing continues to drop. It appears, then, that we now have a golden opportunity to perform these kinds of studies and gain deeper insight into this critical parameter. However, a puzzle remains unsolved. Unfortunately, even the so-called direct estimates of mtDNA MRs fail to minimize selection within the cell (reviewed in [10]). This is because the mtDNA exists in multiple copies per mitochondrion and there are multiple mitochondria per cell (figure 1*a*). As a result, estimates of mtDNA MR are not disentangled from the impact of genetic drift and natural selection acting on mutations that occur, initially appearing in only a single copy of the mtDNA (frequency of 1/*N*, where *N* is the number of mtDNA genomes that will be passed on to the next generation, in the case of germline MRs and to the next cell in the case of somatic MRs). Thus, if two taxa appear to have different MRs (figure 1*b*), this could simply be the result of a different size genetic bottleneck between species or different intracellular selection regimes (figure 1*c*).

## 2. Rules and exceptions: unique features of the mtDNA and its pattern of inheritance

Despite the cartoon depictions in textbooks of mitochondria as isolated ovals, the dynamic structure of this organelle as a network and its capacity to undergo fission and fusion are much more well understood today than even a decade ago [11]. Studies not only in yeast, but in both plants [11] and animals [12], have served to expand our understanding of the morphology of not only the mitochondrion, but the mtDNA it contains [13]. One or more copies of the mtDNA genomes are now known to be complexed with proteins and scattered throughout the matrix (in structures called nucleoids) which serve as sites of DNA replication and transcription for the organelle. Although the number of mtDNA genomes per nucleoid and nucleoid number has been quantified in a few model systems and cell lines (reviewed in [14]), the number of studies is extremely small and the level of variation among cells, tissue types, individuals, populations and species has yet to be characterized. Often, the same few estimates are used repeatedly in the literature (without citation) for back-of-the-envelope calculations, but as we shall discuss below, the actual number of mtDNA in the cell and their tendency to form clusters is of critical importance for understanding how the relative strength of selection and drift act on new mutations when they occur.

The replication of mtDNA is not tied to the replication of nDNA and is not limited to one copy per genome. In addition, it is now known that cells can target certain mtDNA chromosomes for destruction if they do not function properly (‘mitophagy’ [14]). Thus, the number of mtDNA genomes may not only vary from cell to cell but within a given cell can vary over time. While we are debunking common myths, it is worth mentioning that mtDNA genomes can and do recombine (despite dogma to the contrary; [15–17]) and that their capacity for DNA repair is not minimal, as previously thought [18,19]. Overturning these erroneous pillars of the conventional wisdom among biologists is important in general, but especially for the issue of MR estimation in the mtDNA given that recombination and DNA repair represent mechanisms by which mutations may be countered. Another relevant myth about mtDNA is a strict uniparental inheritance. Although uniparental inheritance is, indeed, typical in some systems, episodes of paternal leakage (e.g. [20,21]) and the discovery of doubly uniparental inheritance (reviewed in [22]) are now reported. If one considers the possibility of (even occasional) biparental inheritance as akin to the impact of migration for gene flow between populations, then cases of regular paternal leakage may have a non-trivial impact on the genetic variation in the ‘population’ of mtDNA in the cell. While all heteroplasmy (the presence of more than one allele (or version) of the mitochondrial genome in a given cell or individual) is initially owing to mutation, fluctuations in heteroplasmy level can be explained by not only mutation, but migration, selection and drift acting within the cell as well.

## 3. Why estimating mtDNA ‘mutation rates’ is such a challenge

**Figure 2.**
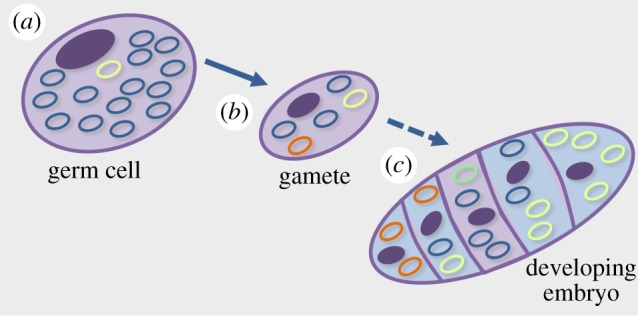
Simplified cartoon of cells (purple) depicting shifting levels of mtDNA heteroplasmy (illustrated by the presence of different colour rings) at several stages. (*a*) Heteroplasmy in a germ cell in a parent organism (represented by blue and yellow rings) can be owing to mutation or biparental inheritance of the mtDNA from the previous generation. (*b*) During gametogenesis (illustrated by the solid arrow), allele frequencies can shift owing to mutation (orange ring), intracellular selection among variants, or chance (genetic drift) owing to the genetic bottleneck that occurs when gametes are formed. (*c*) During development (illustrated by the dashed arrow), allele frequencies in germline (purple) or somatic (blue) cells can also shift as a result of mutation (green ring), intracellular selection or chance.

The existence of mtDNA genomes in multiple copies within each cell (heteroplasmy) means that estimating ‘MRs’ in the mtDNA requires calculating the rates of occurrence of very low frequency (1/*N*) new mutant alleles. The subsequent changes in the frequency of these mutant alleles in the cell over time, which are thought of as part of the MR, actually depend on *other* evolutionary forces, not mutation. Thus, measuring a ‘MR’ *without* the influential effects of migration (leakage, discussed above), selection and drift is a challenge, and partitioning the portion of the ‘MR’ that is observed to these various forces has rarely been discussed, no less tackled. The effective population size (*N*_e_) of mtDNA determines the efficacy of selection within the cell (figure 2*a*), and the size of the genetic bottleneck during gametogenesis and development regulates the power of drift (figure 2*b*). Subsequent to the genetic bottleneck that occurs during gametogenesis, other genetic bottlenecks can occur in somatic tissues, influencing the number and composition of the mtDNA population in cells later on during development as well (figure 2*c*).

The advent of short read/high coverage sequencing platforms has made it possible to sequence the mtDNA to sufficient depth to detect even low-frequency variants, but given the number of mtDNA per nucleoid, the number of nucleoids per cell, and the number of cells per individual, our ability to actually detect new mutations is still extremely limited. This makes it difficult to distinguish between low-frequency variants that might already exist in the ‘population’ and new mutant alleles. Technological advances that result in even more affordable deep sequencing may make this problem tractable, but given that it is not possible to bottleneck the number of mtDNA genomes in the cell completely (to *n* = 1), we cannot eliminate intracellular selection and maximize drift. Thus, estimates of the mtDNA MR will never reflect the ‘true’ rates of mutation owing to unrepaired polymerase faults and DNA damage but are relegated to reflecting the combined interactive nature of multiple evolutionary forces.

In lieu of being able to measure the rate of mutation in mtDNA, let us consider the possibility of measuring the other factors contributing to variation in this composite metric (leakage is discussed above, so the focus will be on selection and drift, hereon). Given the growing body of knowledge about the structure and function of the mitochondrial matrix, it may be possible to empirically estimate factors that contribute to variation in the strength and efficacy of selection, including mtDNA copy number, turnover rates owing to autophagy or recombination rates [23,24]. Indeed, the clustering of mutant alleles in the mitochondrion has been invoked as a mechanism for strengthening the power of selection within the cell (i.e. nucleoids containing mutations could be easily targeted for destruction [25]). Alternatively, cell lines with different mtDNA copy number could be used to establish MA lines or it may be possible to perform selection experiments to generate lineages that vary in mtDNA copy number (and little else). Comparisons of MR differences in lineages with different mtDNA copy number could shed light on the variable selective pressures faced by new mutant alleles within cells. It still will not be *easy* to quantify the role of selection on mtDNA MRs, but it might be possible to investigate empirically.

Of the evolutionary forces combining to determine the perceived rate of mutation, the impact of drift is probably the most readily quantifiable; however, there are two quantities of interest to measure that are often not distinguished in the literature. The first is the effective population size (*N*_e_) of the mtDNA, which depends not only on the number of copies of the genome in the cell, but also on levels of allelic diversity (when present) and the effects of selection on tightly linked loci. The second feature of the cell that regulates the power of genetic drift is the genetic bottlenecks that occur during cell division (either in the germline (figure 2*b*) or the soma (figure 2*c*)). Whereas the *N*_e_ of the mtDNA determines the efficacy of selection during most of the cell cycle, the size of the genetic bottleneck determines the likelihood of fixation or loss of new mutations between generations or from one cell to the next. The *N*_e_ of the mtDNA in a mature cell and the size of the genetic bottleneck during cell division are often conflated and are rarely measured directly, although they could be using deep sequencing methods (with normalization protocols to determine copy number) for the former and cell imaging techniques for the latter.

Even though complete genetic bottlenecking at the level of the mtDNA (*n* = 1) is not possible, experiments comparing the MRs among lineages experiencing different size bottlenecks at the organismal level (*N*_e_ = 1, 10 and 100) show a trend towards higher frequency of mtDNA mutations with reduced effective population size [26]. Studies of cytoplasmic selfish genetic elements (SGEs), in particular, have shown an increase in the frequency of SGEs when selection at the organismal is reduced via small population sizes, but selection within the cell among mtDNA molecules remains (i.e. [27]). In these studies, the authors are able to conclude something about the relative selective impact of various mutations, given their frequency of occurrence—another essential parameter for understanding the impact of drift and selection on mtDNA MRs. The distribution of fitness effects of new mutations has been measured empirically in the mtDNA [28] but is difficult to parse because of tight linkage among mutations and the presence of coding and non-coding regions in plant mtDNAs [29]. The problem of disentangling mutation, selection and drift for estimating mtDNA MRs may thus be relegated to the world of modelling and simulations for the time being (e.g. [30]), while empiricists settle for composite estimates that do not isolate the MR, *per se*, but do provide a more accurate estimate than classical substitution rates calculated based on sequence comparisons alone.

## 4. Is it possible to more accurately estimate mtDNA mutation rates?

Although MA experiments can provide us with MR estimates for nDNA in diploid eukaryotes without worrying about variable levels of influence of intracellular selection and drift, they cannot overcome the challenges outlined above for mtDNA MRs. How can we overcome these obstacles? Haag-Liautard *et al*. [31] outlined a method using the allele frequency distribution (AFD) of new mutant alleles to estimate *N*_e_; however the method is limited at the outset by the sensitivity of the techniques used to detect such mutations (even with deep sequencing, it is difficult to detect mutant alleles when they first occur and are at 1/*N* frequency). The basic idea is to use AFD to quantify the degree of heteroplasmy and, in turn, to estimate the *N*_e_ of the mtDNA. These authors only use data from lines where new mutations were detected, but in principle, *any* fluctuation in heteroplasmic allele frequencies (even in cases where no new mutations occur) could be used to help estimate *N*_e_. The problem, however, is that changes in the AFD over time could be owing to forces other than mutation. For example, consider the case of an organism in which a new mutation has occurred at a particular position that was, initially, observed to be G in all copies of the mtDNA, but now one copy has a T. If, after three generations, the frequency of the mutant T-bearing allele is higher, it is impossible to know if this is because of paternal leakage, genetic drift, intracellular selection or because there were additional G → T mutation events at this locus. Gene conversion events also have the potential to counter the effects of mutation, and this is especially true in the mitochondrion. The advantages of multi-copy genomes in (i) buffering against the selective impact of mutations, (ii) lowering the chance of fixation of new mutations and (iii) providing more templates for recombination or gene conversion have yet to be fully explored, theoretically or empirically. These issues, combined with the sensitivity issue mentioned above, makes current estimates of *N*_e_ based solely on shifts in AFD hard to rely on.

Simulations would be a useful first step to model the relative importance of these factors influencing mtDNA MRs (e.g. [30,32]). One could test a range of mtDNA copy numbers, selective coefficients for new mtDNA mutations, bottleneck sizes and even incorporate practical parameters, such as depth of sequencing to detect new variants and sequencing error rates. A comparative genomics approach might also be useful for testing predictions about patterns of mtDNA MR variation across lineages and species (e.g. [7,33–35]). The widely known MR differences among animal and plant mtDNA [36], for example, are based on estimates from only a few studies of plants which probably painted a misleading picture of mtDNA mutation dynamics that discouraged follow-up studies (but see [37]). Additional intraspecific comparisons could shed light on persistent questions, such as do mtDNA rates vary between sexual and asexual lineages of the same species or among different ploidy levels? In theory, the reproductive mode should have little impact on the mutation dynamics of organellar DNA, but this has yet to be tested empirically with deep sequencing techniques (but see [38]). Given the growing number of exceptions to the previously accepted ‘rules’ of mtDNA inheritance, it may be possible to identify cases where mutation pressures favour or disfavour the evolution of certain features of the mtDNA genome, such as its size, genome content and the evolution of the size of the genetic bottleneck that occurs between generations (e.g. [39]).

Another useful area of inquiry would be to examine differences in mtDNA rates among somatic cells sampled from tissue types with known differences in mean mtDNA number or bottleneck size (e.g. [30,40,41]). Advances in single-cell sequencing and cell biology techniques to isolate organellar components could make this an affordable strategy for deep sequencing mtDNA genomes (while excluding nuclear genomes) for replicate cells with various mtDNA content and/or differential bottleneck sizes during cell division [42]. Because the differentiation of germline and somatic tissues is distinctive among eukaryotic lineages (most obviously between plants and animals, but there are also remarkable differences within these major groups), comparative studies of germline and somatic mtDNA mutation could be very revealing. Investigating MRs in other types of organellar genomes (e.g. the chloroplast genome) would also be illuminating, given that the few extant studies suggest wild variation exists in the mutation profiles of, for example, mitochondrial genomes and those in the chloroplast based on substitution rates [43,44]. Additional empirical estimates of not only mtDNA ‘MRs’, but mtDNA copy number and variation in the size of the genetic bottlenecks during cell division, will be critical for disentangling the interactive effects of mutation, selection and drift on rates of change in the mitochondrial genome [45].

## 5. Conclusion

The rate of mutation in the mtDNA is a foundational parameter in biology. Although, in principle, mtDNA MRs depend only on the rate of replication mistakes and unrepaired DNA damage, the fact that there are multiple copies of the mtDNA genome per mitochondrion and multiple mitochondria per cell means that the fate of a given mutation in the cell also depends on its selective coefficient and the efficacy of selection to act upon that mutation, relative to the likelihood that it will be lost or fixed by genetic drift. Thus, in order to accurately measure mtDNA rates, one must take a synthetic view of this parameter and meet the challenge of estimating its component parts. In addition, it is essential that we obtain not only estimates just for a single genotype from a few model species, but expand our empirical assays to include non-model organisms and multiple genotypes per taxon, in order to understand how mtDNA MRs evolve. Finally, investigating both germline and somatic mtDNA MRs represents a current frontier in the field. While the advent of high-throughput sequencing made direct estimates of nDNA MRs using MA an attainable goal, single-cell sequencing (and even single mitochondrion sequencing [42]) technologies should extend this power to mtDNA MR estimation. Knowing the rate of change in the mtDNA genome is not only a critical component for understanding the function and dysfunction of the mitochondrion [46], it has a wide array of other consequences. In an applied sense, rates of mtDNA change may be correlated with cancer risk and the probability of other mitochondrial diseases [2,47,48]. In terms of basic biology, knowing such rates can inform our understanding of evolution at many levels: the coevolutionary dynamics between proteins encoded in different compartments of the cell [49], the evolution of cell types (including germline and somatic differences; [39]), and the evolution of species differences in life history [50], reproductive mode [51] or patterns of inheritance [52,53].

## Data accessibility

This article has no additional data.
